# Easily applicable predictive score for MPR based on parameters before neoadjuvant chemoimmunotherapy in operable NSCLC: a single-center, ambispective, observational study

**DOI:** 10.1097/JS9.0000000000001050

**Published:** 2024-01-23

**Authors:** Mingming Hu, Xiaomi Li, Haifeng Lin, Baohua Lu, Qunhui Wang, Li Tong, Hongxia Li, Nanying Che, Shaojun Hung, Yi Han, Kang Shi, Chenghai Li, Hongmei Zhang, Zhidong Liu, Tongmei Zhang

**Affiliations:** a Department of Oncology; b Department of Medical Imaging; c Department of Pathology; d Department of Thoracic Surgery, Beijing Chest Hospital, Capital Medical University; e Department of Oncology, Beijing Institute of Tuberculosis and Chest Tumor, Beijing, People’s Republic of China

**Keywords:** major pathological response, neoadjuvant chemoimmunotherapy, nonsmall cell lung cancer, predictive model

## Abstract

**Background:**

Neoadjuvant chemoimmunotherapy (NACI) is promising for resectable nonsmall cell lung cancer (NSCLC), but predictive biomarkers are still lacking. The authors aimed to develop a model based on pretreatment parameters to predict major pathological response (MPR) for such an approach.

**Methods:**

The authors enrolled operable NSCLC treated with NACI between March 2020 and May 2023 and then collected baseline clinical-pathology data and routine laboratory examinations before treatment. The efficacy and safety data of this cohort was reported and variables were screened by Logistic and Lasso regression and nomogram was developed. In addition, receiver operating characteristic curves, calibration curves, and decision curve analysis were used to assess its power. Finally, internal cross-validation and external validation was performed to assess the power of the model.

**Results:**

In total, 206 eligible patients were recruited in this study and 53.4% (110/206) patients achieved MPR. Using multivariate analysis, the predictive model was constructed by seven variables, prothrombin time (PT), neutrophil percentage (NEUT%), large platelet ratio (P-LCR), eosinophil percentage (EOS%), smoking, pathological type, and programmed death ligand-1 (PD-L1) expression finally. The model had good discrimination, with area under the receiver operating characteristic curve (AUC) of 0.775, 0.746, and 0.835 for all datasets, cross-validation, and external validation, respectively. The calibration curves showed good consistency, and decision curve analysis indicated its potential value in clinical practice.

**Conclusion:**

This real world study revealed favorable efficacy in operable NSCLC treated with NACI. The proposed model based on multiple clinically accessible parameters could effectively predict MPR probability and could be a powerful tool in personalized medication.

## Introduction

Highlights

- The efficacy and safety data of neoadjuvant chemoimmunotherapy in operable nonsmall cell lung cancer was demonstrated in a real-world setting with the largest population.
- An applicable model to predicting major pathological response in nonsmall cell lung cancer received neoadjuvant chemoimmunotherapy from baseline clinical-pathology data and routine laboratory examinations before treatment was developed and validated.
- The proposed model could effectively predict major pathological response probability and could be a potential tool in personalized medication.

It is estimated that there were 238 340 new cases of lung cancer in the United States in 2023, with the incidence second only to prostate cancer in men and breast cancer in women; 127 070 deaths from lung cancer, both in men and women, ranked first among all malignant tumors^1^. Nonsmall cell lung cancer (NSCLC) accounts for 85% of all types of lung cancer, of which about 30% are in the early stage at the first diagnosis^2,3^. Complete surgical resection remains the recommended therapeutic modality for these patients in the curable stage. Neoadjuvant therapy is a systemic antineoplastic therapy performed preoperatively with the purpose to degrade the tumor stage and increase the chance of complete resection, reduce micrometastases, and improve the tolerance to facilitate subsequent operations^4^. However, neoadjuvant chemotherapy only can increase survival rate by about 5%, and there is an urgent need for new treatment modalities to improve survival^5^. In recent years, a number of prospective clinical trials have shown that immune checkpoint inhibitors (ICIs) combined with chemotherapy produced better major pathological response (MPR) and pathological complete response (pCR) than neoadjuvant chemotherapy alone, and preliminary results have indicated that it can translate into survival benefit^6–10^. Therefore, the primary study endpoint of phase II and phase III clinical studies in the neoadjuvant stage is MPR or pCR^11,12^.

However, approximately only one-third of the patients benefit from ICIs. Until now, there are no recommended predictive markers to screen potential candidates in neoadjuvant settings^13^. At present, the companion diagnostics approved for ICIs in advanced NSCLC, such as PD-L1 expression through immunohistochemistry, tumor mutational burden (TMB), and microsatellite instability (MSI), do not perfectly distinguish between those who respond and those who do not. The determination of TMB/MSI was complicated and did not reach a good consensus in terms of the clinical application^14^. All of these could restrict some patients from accessing ICIs even though they could benefit from them, and it also does not accurately identify the patients most likely to respond. Given the potential hazard of ineffective treatment in a disease, which is typically characterized by aggressive tumor growth and then the loss of operation, there is an urgent need to develop a risk-scoring system to identify the right candidates for neoadjuvant chemoimmunotherapy (NACI). Several predictive models based on dynamic medical image mining, immune-related histological phenotype in tumor biopsy, and liquid immune cell profiling have been reported. While, models based on tumor computational images always rely on changes in the radiomic texture of CT patterns before and after immunotherapy, and thus cannot help us perform stratification before treatment^15,16^. A vast amount of omics data are currently available for biomarker research, such as histological phenotype in tumor microenvironment, liquid immune cell profiling, and multigene panel sequencing, but their use is restricted because of the cost and complex processes as well as the relatively more demands for tissue samples^17–21^. Hence, the exploitation of novel, effective, and applicable biomarkers at a low-cost to select suitable patients who could benefit from NACI is necessary and meaningful.

By comparison, peripheral blood-related biomarkers have unique advantages of noninvasiveness, and convenient acquisition. At the same time, peripheral blood can provide a comprehensive immune status of the host, and can monitor the effect dynamically and longitudinally, therefore such results are frequently used in biomarker screening of ICI research^22^. It is expected that classical prognostic variables such as neutrophil-to-lymphocyte ratio (NLR) or serum lactate dehydrogenase (LDH) have been explored as potential predictors of response in a variety of solid tumors, mainly in patients with advanced disease^23^. However, these routinely available peripheral blood markers, such as total cell counts and ratios, biochemistry, coagulation, and tumor markers have not been fully explored in neoadjuvant settings.

In this manuscript, we reported the efficacy and safety data from a NACI cohort conducted in the real world with the largest population, to my furthest knowledge. Sequentially, a predictive model for MPR was developed and validated by virtue of clinical-pathological factors and peripheral blood markers, both of which were routinely examined before the initiation of NACI before the treatment start. The developed model is likely to be a weakly supervised, easy-accessible, low-cost tool for NSCLC management.

## Methods

### Ethics standards

This observational cohort study was conducted at a single center and adhered to the ethical guidelines outlined in the Declaration of Helsinki. The study was approved by our institution’s review board (Approval Number: YNLX-2022-015). Due to the observational study design, the ethics committee approved a waiver of written informed consent. All procedures conducted in our study followed the criteria outlined in REMARK^24^ (Supplemental Digital Content 1, http://links.lww.com/JS9/B754).

### Study design and patient cohort

It was an ambispective (i.e. containing both retrospective and prospective stages of data collection), observational study designed to evaluate the effectiveness, treatment patterns, and safety of NACI in patients with operable NSCLC in routine clinical practice in our hospital. Data from patients who started treatment between 1 March 2020 and 30 July 2022, were retrospectively collected; data were then prospectively collected from 1 August 2022 to 1 October 2023. The ambispective design had no impact on the data collection process or the study methods that would require a separate analysis of the data collected retrospectively and prospectively.

Patients with NSCLC in our hospital were included: (1) pathologically proved, (2) clinical TNM staged I–III, (3) radical resection after neoadjuvant immunochemotherapy, and (4) sufficient organ function, including blood cell count, coagulation parameters, physical score, liver, and kidney function. Patients were excluded from this study when any of the following criteria were met: (1) neoadjuvant immunotherapy/chemotherapy alone, (2) previous radiotherapy, (3) neoadjuvant targeted therapy, (4) locally endoscopic therapy before surgery, (5) chronic viral infections, such as HBV, HCV, or HIV infection, (6) accompanied with active infection requires anti-infective treatment, (7) history of malignant tumor within 5 years, (8) pre-existing autoimmune diseases, (9) pre-existing allergic diseases, and (10) history of solid organ transplantation. Patients’ clinical characteristics, including age, sex, smoking status, BMI, TNM stage, lesion location, pathology, and information regarding their laboratory tests, medication, and operation were gathered from the electronic database.

### Biomarker measurement and data collection

To assess organ function, patients underwent routine peripheral blood tests within 1 week prior to treatment. In these laboratory data, coagulation tests and blood cell counts, along with some derived parameters, NLR, platelet-to-lymphocyte ratio (PLR), lymphocyte-to-monocyte ratio (LMR) were collected in this study. Some other parameters that have been reported as prognostic markers to programmed death-1 (PD-1) inhibitors were also extracted from database in this study, including LDH, C-reactive protein (CRP), albumin (ALB), carcino-embryonic antigen (CEA), and cytokeratin 19 fragment 21-1 (cyfra21-1)^25–29^.

Tumor samples were obtained through tissue biopsy (bronchoscopy or lung puncture) and the expression of programmed death ligand-1 (PD-L1) in tumor cells was conventionally detected by immunohistochemical staining (Daco22C3). One hundred tumor cells were counted microscopically and the expression levels of PD-L1 protein were expressed according to the tumor proportion score (TPS). The percentage of stained cells <1%, 1–49%, ≥50% was defined as negative, low expression, and high expression, respectively^30^.

### Treatment evaluation

All patients received adequate preoperative imaging, which consisted of chest/abdomen CT scan with contrast, MRI of the brain, ultrasound examination of superficial lymph nodes/abdomen and ECT of bone, while, positron emission tomography/CT scan, invasive staging of the mediastinal lymph nodes using either endobronchial ultrasound or mediastinoscopy, if necessary. The clinical stage was determined according to the eighth edition TNM stage classification of the International Association for the Study of Lung Cancer (IASLC)^31^. The antitumor response was evaluated on the basis of the Response Evaluation Criteria in Solid Tumors version 1.1 (RECIST v1.1) after every two treatment courses. Tumor responses were classified as complete response (CR) with definition of disappearance of all target lesions, partial response (PR, at least a 30% decrease in the sum of diameters of target lesions, taking as reference the baseline sum diameters), stable disease (SD, neither sufficient shrinkage to qualify for PR nor sufficient increase to qualify for PD (progressive disease), taking as reference the smallest sum diameters while on study), or PD (at least a 20% increase in the sum of diameters of target lesions, taking as reference the smallest sum on study). ORR was the percentage of patients who achieve CR or PR according to RECIST criteria^32^.

Pathological response of neoadjuvant therapy was evaluated by two qualified pathologist based on H&E staining. MPR refers to the induction and elimination of residual tumor from tumor pathology after neoadjuvant therapy in ≤10%. pCR is defined as complete tumor regression induced by neoadjuvant therapy without pathological residual tumor^33^. Adverse events were evaluated according to the National Cancer Institute General Toxicity Standard version 3.0^34^.

### Model development and validation

Given the inevitable problem of missing data, we used a multiple-imputation approach. The imputation method involved in continuous variables was predictive mean matching and the distributions of raw data and imputed values were viewed by density plots.

**Figure 1:**
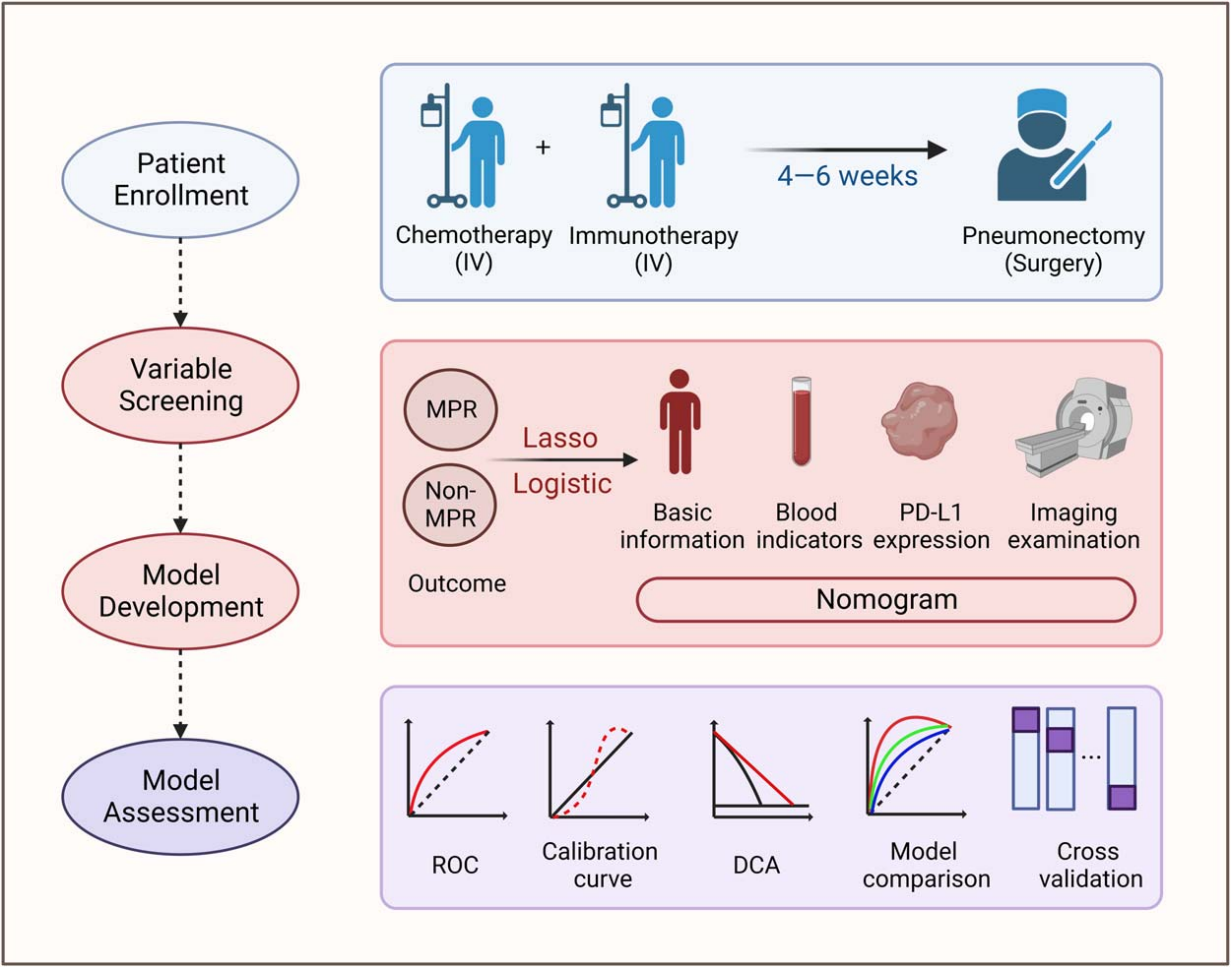
The overall experimental design of this study. DCA, decision curve analysis; IV, intravenous injection; MPR, major pathological response; PD-L1, programmed death ligand-1; ROC, receiver operating characteristic. Created with BioRender.com.

We used the patient cohort from March 2020 to May 2023 as the training set, while June 2023 to October 2023 as the validation set. We performed a univariate Logistic regression and Lasso regression to screen variables, respectively, and further fitted them by multivariate Logistic regression to determine factors associated with MPR based on the minimum Akaike information criterion (AIC) value. Factors found to be independent were utilized to create nomogram models. We plotted receiver operating characteristic curves (ROC) and calculated the area under the curve (AUC) to describe their discriminatory power. In addition, to assess the agreement between the observed and predicted probabilities, we created calibration plots using bootstrapping (1000 resamplings). A *P*-value greater than 0.05 for the Hosmer–Lemeshow test indicates good agreement. To assess the utility of these findings in the clinical setting, we performed decision curve analysis (DCA). This study intends to use 10-fold 200 times cross-validation to verify the reproducibility of the model in the development process, and calculate the mean values of various indicators including AUC, R2, discrimination index, *U* test, Brier score, maximum offset, and minimum offset. Finally, the generalization evaluation of the model was performed in the validation set, ROC, calibration curve, and DCA curve were plotted, and the above indicators were calculated. The overall experimental design was depicted in Figure 1.

### Statistical analysis

This study used R (version 4.2.0) and GraphPad Prism (version 8.0) to analyze and collate the data. Means±SD and medians plus quartiles were used to represent normally and non-normally distributed continuous variables, respectively. Categorical variables were presented as absolute counts and percentages. Fisher’s exact test/χ^2^ test and Kruskal–Wallis test were used to analyze categorical and continuous variables, respectively. Pearson χ^2^ test or Fisher exact test was used to compare differences in response and clinical benefit between the groups. Univariate Logistic regression analysis was used to screen for the potentially important factors, and then multivariate Logistic regression analysis to identify the independent predictors, which could differentiate MPR group from the whole cohort. Moreover, the further significance of nomogram factors was identified using LASSO regression analysis. *P*-values less than 0.05 were considered statistically significant, and all hypothesis tests were two-sided. The software packages involved in the study were ‘mice’, ‘plyr”, ‘MASS’, ‘glmnet’, ‘rms’, ‘rmda’, and ‘caret’.

## Results

### Patient cohort

**Figure 2:**
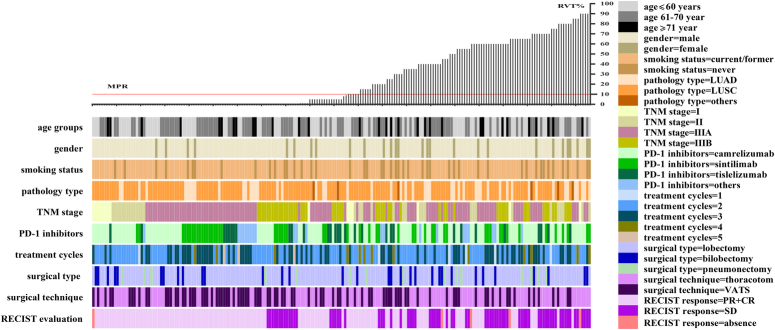
Waterfall plot of pathological response of neoadjuvant chemoimmunotherapy of the 206 patients. Each bar represents one patient. The upper rows show clinical and pathological characteristics. CR, complete response; LUAD, lung adenocarcinoma; LUSC, lung squamous cell carcinoma; MPR, major pathological response; PD-1, programmed death-1; PR, partial response; RECIST, response evaluation criteria in solid tumors; RVT, residual viable tumor; SD, stable disease; TNM, tumor node metastasis; VATS, video-assisted thoracoscopic surgery.

A total of 206 patients were enrolled in the final cohort between March 2020 and May 2023 (Fig. S1, Supplemental Digital Content 2, http://links.lww.com/JS9/B755). Patients’ baseline clinical characteristics are displayed in Figure 2 and more details are listed in Table 1, with a median age of 62.5 years (range 57.0–67.8 years) and the majority of patients were male (178, 86.4%). According to the IASLC eighth edition TNM classification for lung cancer, 156 (75.7%) patients had stage III disease, including 104 and 52 patients with stage IIIA and IIIB, respectively. One hundred and fifty-nine (77.2%) patients were former or current smokers and 47 (22.8%) patients were nonsmokers. In 206 patients, lung squamous cell carcinoma (LUSC) was the most common pathological type (*n*=149, 72.3%), while 51 (24.8%) cases were lung adenocarcinoma (LUAD). The most common site of tumor was the right upper lobe (RUL, *n*=60, 29.1%), followed by the left upper lobe (LUL, *n*=50, 24.3%), left lower lobe (LLL, *n*=39, 18.9%), right middle lobe (RML, *n*=32, 15.5%), right lower lobe (RLL, *n*=22, 10.7%), and left main bronchus (LMB, *n*=3, 1.5%).

**Table 1:** Patient baseline characteristics in MPR and non-MPR groups.

Characteristic	Overall (*N*=206)	Non-MPR (*N*=96)	MPR (*N*=110)	*P*
Age [median (IQR)]	62.5 [57.0–67.8]	63.5 [56.0–68.0]	62.0 [58.0–67.0]	0.93
Gender (%)				
Male	178 (86.4)	77 (80.2)	101 (91.8)	0.03
Female	28 (13.6)	19 (19.8)	9 (8.2)	
Smoking status (%)				
Never	47 (22.8)	29 (30.2)	18 (16.4)	0.03
Current/former	159 (77.2)	67 (69.8)	92 (83.6)	
Pathological type (%)				
LUSC	149 (72.3)	63 (65.6)	86 (78.2)	0.06
non-LUSC	57 (27.7)	33 (34.4)	24 (21.8)	
TNM stage (%)				
I–II	50 (24.3)	22 (22.9)	28 (25.5)	0.67
IIIA	104 (50.5)	47 (49.0)	57 (51.8)	
IIIB	52 (25.2)	27 (28.1)	25 (22.7)	
ypTNM stage (%)				
ypT_0_N_0_M_0_		0 (0.0)	76 (100.0)	
ypT_0_N_1-2_M_0_		0 (0.0)	7 (100.0)	
ypT_1_N_0_M_0_		36 (61.0)	23 (39.0)	
ypT_1_N_1-2_M_0_		43 (91.5)	4 (8.5)	
ypT_2-3_N_0_M_0_		9 (100.0)	0 (0.0)	
ypT_2-3_N_1-2_M_0_		8 (100.0)	0 (0.0)	
Neoadjuvant treatment cycles (%)				
≤2	114 (55.3)	53 (55.2)	61 (55.5)	1.00
>2	92 (44.7)	43 (44.8)	49 (44.5)	
PD-1 inhibitors				
Camrelizumab	101 (40.0)	49 (51.0)	52 (47.3)	0.88
Sintilimab	53 (25.7)	25 (26.0)	28 (25.5)	
Tislelizumab	30 (14.6)	12 (12.5)	18 (16.4)	
Others	22 (10.7)	10 (10.4)	12 (10.9)	
BMI [median (IQR)]	23.9 [21.9–26.5]	24.2 [22.4–26.7]	23.4 [21.5–26.4]	0.25
PD-L1 [median (IQR)]	10.0 [1.0–70.0]	1.5 [0.0–42.5]	40.0 [1.0–80.0]	0.00
Cyfra21-1 [median (IQR)]	5.6 [3.3–17.0]	5.4 [3.3–18.6]	5.8 [3.6–15.8]	0.92
LDH [median (IQR)]	163.0 [145.0–189.5]	166.5 [146.8–188.0]	161.5 [143.2–192.2]	0.80
CRP [median (IQR)]	5.8 [1.7–15.3]	5.8 [1.8–13.8]	5.6 [1.7–19.0]	0.97
CEA [median (IQR)]	3.2 [2.1–6.0]	3.6 [2.2–7.3]	3.0 [2.1–5.0]	0.07
PT [median (IQR)]	11.4 [10.8–11.9]	11.3 [10.6–11.8]	11.6 [11.0–12.2]	0.00
INR [median (IQR)]	1.0 [0.9–1.0]	1.0 [0.9–1.0]	1.0 [0.9–1.1]	0.06
PT% [median (IQR)]	84.7 [73.2–96.9]	86.3 [77.4–100.6]	80.2 [71.7–92.8]	0.01
PTR [median (IQR)]	1.0 [0.9–1.0]	1.0 [0.9–1.0]	1.0 [1.0–1.0]	0.01
APTT [median (IQR)]	28.3 [25.7–31.3]	27.8 [25.2–30.4]	28.6 [26.5–31.8]	0.05
FIB(S) [median (IQR)]	6.8 [5.6–8.3]	6.6 [5.5–7.9]	7.0 [5.7–8.4]	0.12
FIB [median (IQR)]	3.4 [2.8–4.4]	3.4 [2.8–4.3]	3.4 [2.8–4.6]	0.80
TT [median (IQR)]	18.7 [17.9–19.4]	18.8 [17.8–19.4]	18.6 [17.9–19.4]	0.64
D-Dimer [median (IQR)]	0.4 [0.3–0.6]	0.4 [0.3–0.6]	0.4 [0.3–0.6]	0.83
WBC [median (IQR)]	7.3 [6.1–8.6]	7.1 [6.1–8.6]	7.4 [5.8–8.5]	0.90
LY% [median (IQR)]	25.4 [20.7–31.0]	24.6 [19.2–29.2]	26.0 [21.5–31.6]	0.06
M0N0% [median (IQR)]	6.3 [5.5–7.6]	6.3 [5.5–7.3]	6.3 [5.5–7.6]	0.59
NEUT% [median (IQR)]	64.4 [58.9–70.4]	65.4 [60.5–71.8]	63.3 [58.1–68.7]	0.01
RBC [median (IQR)]	4.6 [4.2–4.9]	4.5 [4.2–4.8]	4.6 [4.3–4.9]	0.39
HGB [median (IQR)]	137.5 [126.0–147.8]	137.0 [127.8–146.0]	138.0 [124.2–149.0]	0.87
HCT [median (IQR)]	40.8 [37.8–43.9]	40.4 [38.3–43.6]	41.0 [37.5–44.2]	0.84
MCV [median (IQR)]	89.6 [86.9–92.9]	89.8 [86.9–92.8]	89.4 [87.0–92.7]	0.34
MCH [median (IQR)]	30.3 [29.3–31.5]	30.4 [29.3–31.4]	30.1 [29.2–31.6]	0.67
MCHC [median (IQR)]	336.0 [327.0–343.0]	337.0 [328.0–344.0]	335.0 [325.2–342.0]	0.11
RDW-CV [median (IQR)]	12.6 [12.0–13.2]	12.4 [12.0–13.2]	12.6 [12.1–13.2]	0.24
PLT [median (IQR)]	239.0 [198.0–291.2]	235.5 [197.8–281.5]	245.0 [198.2–296.2]	0.58
MPV [median (IQR)]	10.0 [9.4–10.8]	9.9 [9.3–10.6]	10.1 [9.6–11.0]	0.09
P-LCR [median (IQR)]	25.0 [20.4–31.5]	23.3 [19.4–29.0]	25.7 [21.7–32.5]	0.02
PDW [median (IQR)]	11.4 [10.1–13.2]	10.9 [9.8–12.4]	11.7 [10.5–14.1]	0.04
EOS% [median (IQR)]	2.4 [1.5–3.4]	2.2 [1.5–2.8]	2.7 [1.5–3.8]	0.02
BASO% [median (IQR)]	0.4 [0.3–0.6]	0.4 [0.3–0.6]	0.5 [0.3–0.6]	0.63
RDW-SD [median (IQR)]	41.3 [39.3–43.8]	41.0 [39.3–43.6]	41.3 [39.4–43.8]	0.72
NLR [median (IQR)]	2.5 [1.9–3.4]	2.6 [2.1–3.8]	2.4 [1.8–3.2]	0.04
LMR [median (IQR)]	4.0 [3.0–5.2]	3.7 [2.8–5.0]	4.1 [3.2–5.3]	0.14
PLR [median (IQR)]	9.5 [6.9–13.5]	10.2 [7.0–13.9]	9.3 [6.5–12.6]	0.22

APTT, partial thromboplastin time; BASO%, basophilic cells percentage; CEA, carcino-embryonic antigen; CRP, C-reactive protein; EOS%, eosinophilic cells percentage; FIB(S), fibrinogen time; FIB, fibrinogen content; HCT, hematocrit; HGB, hemoglobin; INR, international normalized ratio; LDH, lactate dehydrogenase; LMR, lymphocyte-to-monocyte ratio; LUSC, lung squamous cell carcinoma; LY%, lymphocyte percentage; M0N0%, monocyte percentage; MCH, mean corpuscular hemoglobin; MCHC, mean corpuscular hemoglobin concentration; MCV, mean corpuscular volume; MPR, major pathological response; MPV, mean platelet volume; NEUT%, neutrophilic granulocyte percentage; NLR, neutrophil-to-lymphocyte ratio; PD-1, programmed death-1; PDW, platelet distribution width; P-LCR, platelet large cell ratio; PLR, platelet-to-lymphocyte ratio; PLT, platelet; PT%, prothrombin percentage; PT, prothrombin time; PTR, prothrombin ratio; RBC, red blood cell; RDW-CV, red blood cell distribution-coefficient of variation; RDW-SD, red cell distribution width-standard deviation; TNM, tumor node metastasis; TT, thrombin time; WBC, white blood cell.

### NACI regimen

All included patients received PD-1 inhibitor and platinum-based chemotherapy, including taxanes, pemetrexed, and gemcitabine, followed by surgical resection within 4–6 weeks after neoadjuvant therapy. Patients were treated intravenously with one of the following PD-1 inhibitors as neoadjuvant immunotherapy: camrelizumab, sintilimab, tislelizumab, toripalimab, palizumab, and nivolumab. More than half of the patients (*n*=108, 52.4%) received two cycles of drug, followed by three cycles (*n*=76, 36.9%), four cycles (*n*=13, 6.3%), one cycle (*n*=6, 2.9%), and five cycles (*n*=3, 1.5%). In addition, among these 206 patients, 159 (77.2%), 32 (15.5%), and 15 (7.3%) underwent lobectomy/bilobectomy/pneumonectomy, respectively, through video-assisted thoracoscopic surgery (VATS, *n*=90, 43.7%) or thoracotomy (*n*=116, 56.3%, Fig. 2). All cases achieved R0 resection.

### Peripheral blood and biomarkers

For patients treated with NACI, 176 samples were investigated for PD-L1 expression in cancer tissues using immunohistochemistry (IHC). Forty-seven (26.7%) were negative for PD-L1 expression (TPS=0), 69 (39.2%) were positive for weak expression (TPS 1–49%), and 60 (34.1%) were positive for strong expression (TPS ≥50%). In 206 patients undergoing surgery, complete blood count, coagulation parameters, LDH, CRP, and CEA were missing to varying degrees (Fig. S2, Supplemental Digital Content 2, http://links.lww.com/JS9/B755), and these test parameters and loss of PD-L1 expression in tissue were multiply imputed using a predictive mean matching method with five imputations and five iterations, and density plots (Fig. S3, Supplemental Digital Content 2, http://links.lww.com/JS9/B755) showed that imputation was better for each variable.

### Treatment outcomes and differences between groups

In this study, 201 NSCLC were evaluated with radiological imaging after receiving NACI, among whom, 1, 128 and 72 patients achieved CR, PR, and SD, respectively, with ORR of 64.2% (129/201) and no disease progression. Histopathological evaluation was performed in all patients after surgery, pCR was achieved in 36.9% (76/206) and MPR in 53.4% (110/206). MPR and pCR accounted for 69.8% (90/129) and 49.6% (64/129), respectively, in patients who achieved PR by imaging assessment, respectively. In patients who were classified in SD group, MPR and pCR accounted for 26.4% (19/72) and 15.3% (11/72), respectively. One hundred forty-six patients (70.9%) reported treatment-related adverse events, most of which were classified as grades 1–2. Grades 3–4 treatment-related adverse events were experienced by 42 patients (20.4%), with the most common being decreased white cell count in 19 patients (10.7%). The immune-related adverse effects (irAE) was occurred in 60 (29.1%) patients, the most common adverse event was reactive cutaneous capillary endothelial proliferation (RCCEP, 28/206), all of which were graded 1–2, followed closely by rash (22/206) and pneumonia (7/206). Grades 3–4 irAE were reported with maculopapular rash in 16 patients (7.8%), pneumonia in five patients (2.4%), diarrhea in two patients (1.0%), thyroiditis in one patient (0.5%) and myositis in one patients (0.5%). In this cohort, one patient experienced grade 3 rash and thyroiditis simultaneously, while another patient experienced grade 3 rash and pneumonia simultaneously. Grade 5 toxicities were never observed during the neoadjuvant treatment courses. We did not find any correlation between the irAE and the values of blood markers (Table S1, Supplemental Digital Content 2, http://links.lww.com/JS9/B755).

The baseline comprehensive table showed differences in the distribution of patients’ baseline characteristics between the MPR and non-MPR groups, with statistical differences found for prothrombin time (PT, *P*=0.004), prothrombin time percentage (PT%, *P*=0.006), prothrombin ratio (PTR, *P*=0.008), neutrophil percentage (NEUT%, *P*=0.013), platelet large cell ratio (P-LCR, *P*=0.016), platelet distribution width (PDW, *P*=0.041), eosinophils percentage (EOS%, *P*=0.023), sex (*P*=0.026), smoking history (*P*=0.028), PD-L1 expression (*P*=0.0002). While the parameters related to the course of treatment, neither treatment cycles nor different PD-1 inhibitors, affect MPR significantly (Table 1). Because stage III is a very heterogeneous population, separate analyses was done and it showed that patients with IIIA and IIIB had similar benefits in different subgroups (Table S2, Supplemental Digital Content 2, http://links.lww.com/JS9/B755).

### Variable screening and model building

**Figure 3:**
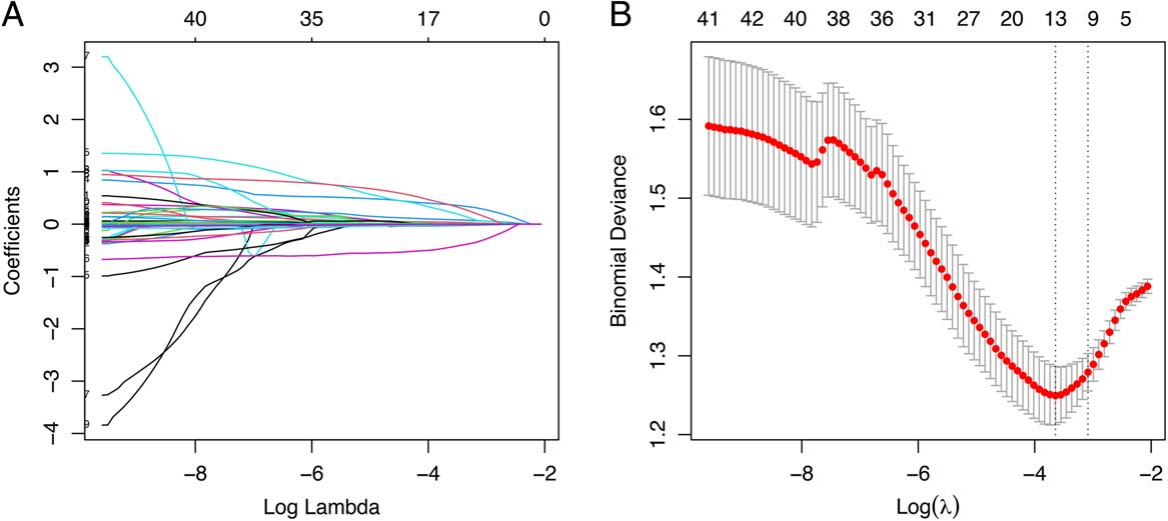
Variable exploration and screening by Lasso regression. (A) Lasso regression model was employed to identify potential parameters from 43 variables, as evidenced by their coefficient profiles in this cohort. (B) Partial likelihood deviance analysis was constructed to determine significant combination of these variables using the Lasso regression model. The red dots represent detailed partial likelihood of deviance values while gray lines indicate standard error.

**Figure 4:**
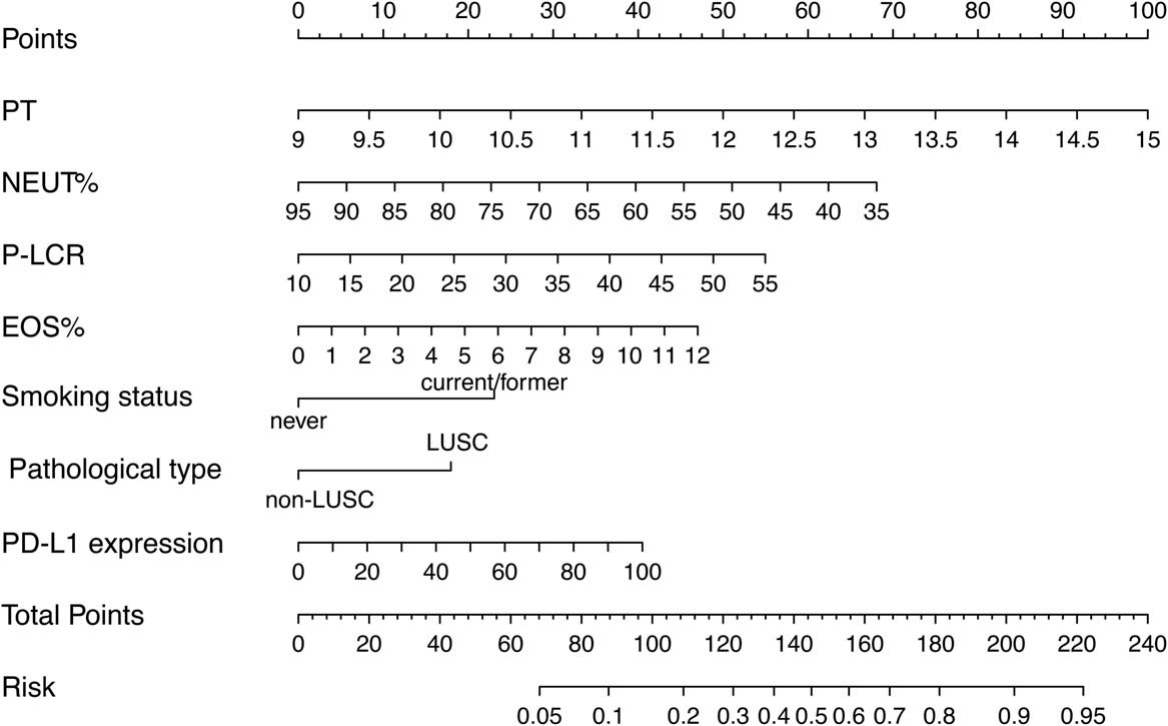
Nomogram was developed based on clinical-pathological factors and peripheral blood parameters at baseline to predict probability of MPR. EOS, eosinophilic cells; LUSC, lung squamous cell carcinoma; NEUT, prothrombin time; PD-L1, programmed death ligand-1; PT, prothrombin time; P-LCR, platelet large cell ratio.

We screened a total of 43 variables using Logistic regression and Lasso regression, respectively. Univariate Logistic regression showed that PT, PT%, NEUT%, P-LCR, PDW, EOS%, sex, smoking status, and PD-L1 expression were associated with MPR (Table S3, Supplemental Digital Content 2, http://links.lww.com/JS9/B755). Multivariate Logistic regression further fitted the model and identified six independent predictors of MPR: PT, NEUT%, P-LCR, EOS%, smoking status, and PD-L1 expression. Taking into account the question of collinearity between these variables, we used two variable screening method to develop the model. Lasso regression established 10 variables associated with MPR: PT, NEUT%, MCHC, P-LCR, PDW, EOS%, sex, smoking status, pathological type, and PD-L1 expression; multivariate Logistic regression results showed that PT, NEUT%, P-LCR, EOS%, smoking status, pathological type, and PD-L1 expression fitted best (Fig. 3A and B). Logistic and Lasso models had AIC values of 263.63 and 261.97, respectively, so seven variables in the Lasso regression were selected as final predictors. According to the variables mentioned above, we constructed a prognostic model while drawing a nomogram to predict the probability of MPR in NSCLC receiving NACI (Fig. 4).

### Model evaluation and validation

**Figure 5:**
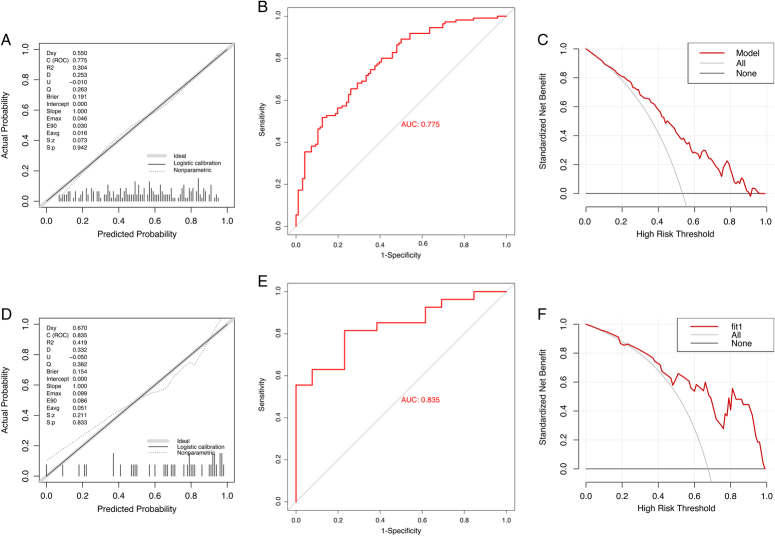
Evaluation of the effectiveness of the nomogram. (A) Calibration plots of the combination model. (B) The ROC curves of the combination model. (C) Decision curve analysis of the combination model. (D) Calibration plots in the external validation dataset. (E) The ROC curves in the external validation dataset. (F) Decision curve analysis in the external validation dataset.

First of all, the calibration curves of the nomogram model demonstrated a satisfied agreement between the predicted and observed results in this cohort with a *P*-value of 0.942, implying an almost identical probability to the actual one (Fig. 5A). The ROC curve showed an AUC value of 0.775 (95% CI: 0.712–0.838), which indicated fine discrimination (Fig. 5B). The decision curve showed that, especially when the threshold probability was >0.2, the positive net benefit of this model for MPR probability prediction was more obvious compared with the ‘all’ or ‘none’ scheme (Fig. 5C). In this study, 10-fold cross-validation was used to evaluate the repeatability of the model. We describes the mean values of various indicators of the model after all datasets (AUC=0.775) and 200 repeated internal validation in Table S4 (Supplemental Digital Content 2, http://links.lww.com/JS9/B755) (AUC=0.746). A total of 40 patients were included as the validation cohort from June 2023 to October 2023. The model also showed good calibration, discrimination (AUC=0.835), and clinical decision-making ability in the validation set (Fig. 5D-F and Table S4, Supplemental Digital Content 2, http://links.lww.com/JS9/B755).

In addition, we used the seven variables described above to construct a predictive model using ORR as an outcome measure in a cohort of 201 patients who underwent radiographic assessment. At the same time, we plotted ROC curve (AUC=0.705, Fig S4a, Supplemental Digital Content 2, http://links.lww.com/JS9/B755) and histogram of difference in total point score (TPS) between ORR group and non-ORR group (Fig S4b, Supplemental Digital Content 2, http://links.lww.com/JS9/B755), although they were less powerful than the above model, they still showed some predictive ability.

### Comparison with different nomograms

**Figure 6:**
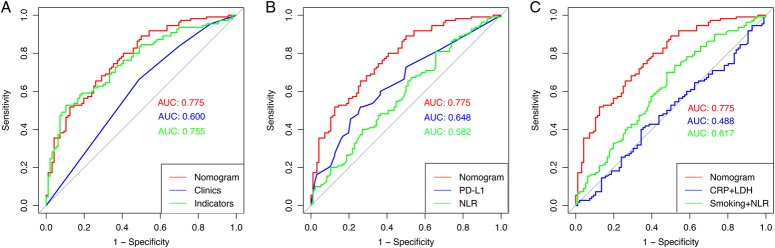
The contrast of ROC indicating MPR based on different combination models. (A) The comparison between our model with clinical-pathological or peripheral blood indicators alone. (B) The comparison between our model with NLR or PD-L1. (C) The comparison between our model with other two models developed in advanced NSCLC, that is, CRP combined with LDH and NLR combined smoking status, respectively. AUC, the area under the curve; CRP, C-reactive protein; LDH, lactate dehydrogenase; NLR, neutrophil-to-lymphocyte ratio; PD-L1, programmed death ligand-1.

We used AUC values to compare the discriminatory power of different models or variables in predicting MPR, as displayed in Figure 6. The AUC values of the models constructed by our combined model, clinical-pathological variables and peripheral blood indicators were 0.775, 0.600, and 0.755, respectively, while the AUC values of PD-L1 expression and NLR were 0.648 and 0.582 (Fig. 6A and B). Meanwhile, our model was compared with other two models, which had been reported as prognostic biomarkers in advanced NSCLC treated with ICIs^35,36^. The result showed that, the AUC values of CRP combined with LDH, and smoking status combined with NLR were 0.488 and 0.617, respectively (Fig. 6C).

### Performance of the nomogram in stratifying patient risk

**Figure 7:**
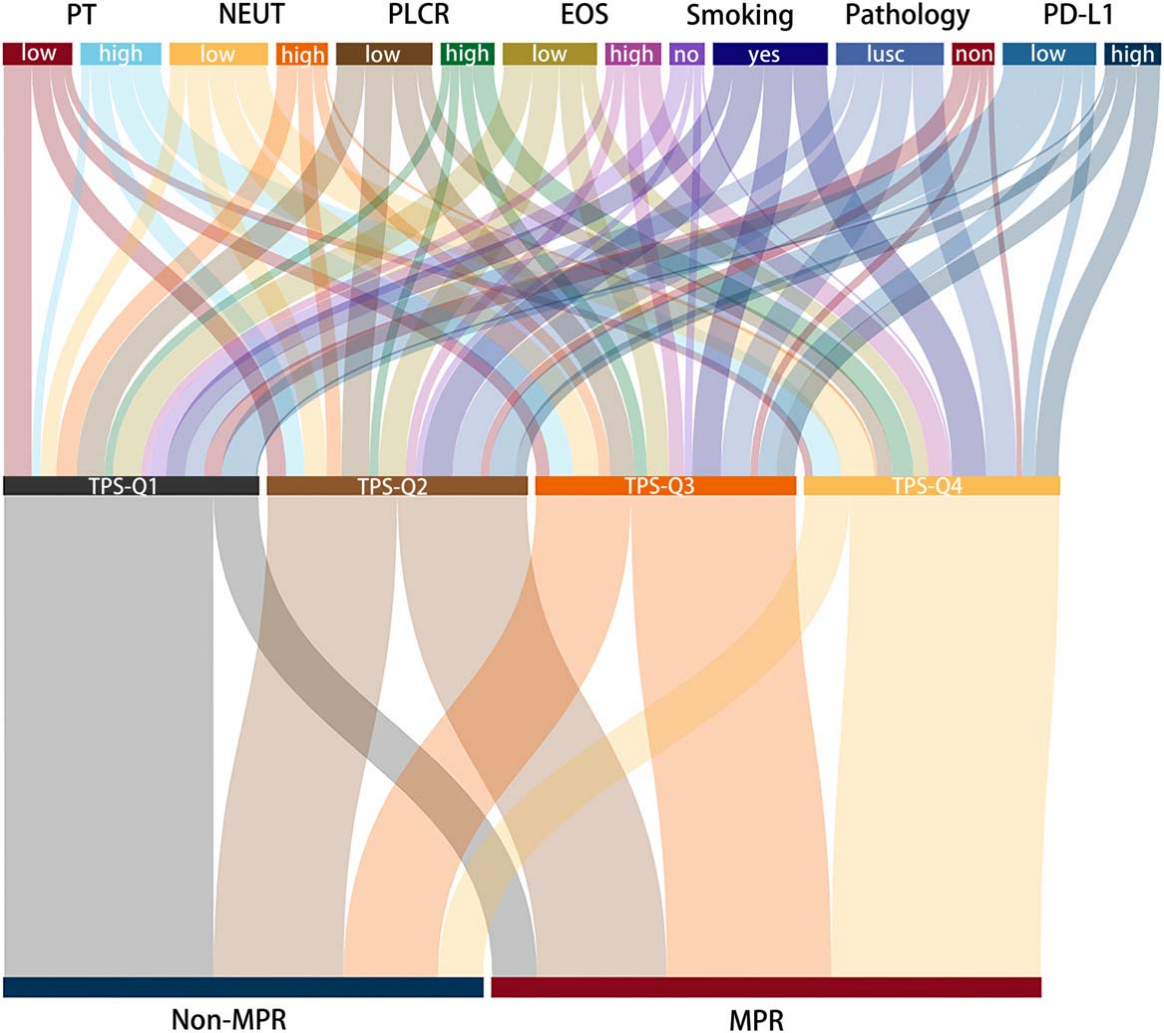
The nomogram-based total score was presented a Sankey diagram. EOS, eosinophilic cells; LUSC, lung squamous cell carcinoma; MPR, major pathological response; NEUT, prothrombin time; PT, prothrombin time; P-LCR, platelet large cell ratio; PD-L1, programmed death ligand-1; TPS, total point score.

Finally, seven variables were included in the current nomogram, then participants were divided into four groups according to nomogram-based TPS quartiles. In comparison to lower quartile, increased TPS quartiles was correlated with favorable MPR probability (Fig. 7). The MPR percentage across increasing quartiles of TPS were 17.6, 50, 63.5, 82.4%, respectively (Fig. S5, Supplemental Digital Content 2, http://links.lww.com/JS9/B755).

### Typical case image

**Figure 8:**
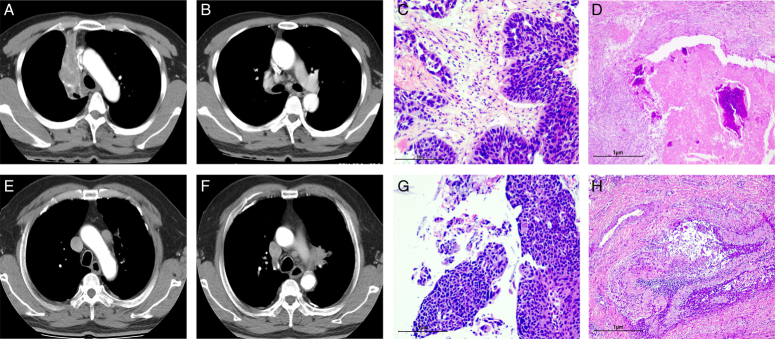
A–D A 47-year-old male LUSC patient with PD-L1 expression=1%, PT=11.7s, NEUT%=69.8%, P-LCR=36%, EOS%=0.8%, without smoking history. (A) A mass in the upper lobe of the right lung near the hilar region, with obstructive pulmonary atelectasis. (B) Enlarged lymph nodes at 4R station. (C) LUSC confirmed by tracheoscopic biopsy (100×). (D) Surgical specimens demonstrated 70% residual viable tumor in tumor bed (40×). E–H A 60-year-old male LUSC patient with PD-L1 expression=20%, PT=11.5s, NEUT%=54.8%, P-LCR=29.5%, EOS%=5.4%, with tobacco use. (A) A mass in the upper lobe of the left lung near the hilar region. (B) Enlarged lymph nodes at six station. (C) LUSC confirmed by tracheoscopic biopsy (100×). (D) Surgical specimens demonstrated cPR (40×).

Case 1: A 47-year-old male LUSC patient with a TPS of 126.84, cT2N2M0 in the upper lobe of the right lung, derived 70% residual viable tumor cell after three cycles of NACI and radical resection (camrelizumab+carboplatin+albumin-bound paclitaxel) (Fig. 8A–D).

Case 2: A 60-year-old male LUSC patient with a TPS of 181.37, cT2N2M0 in the upper lobe of the left lung achieved MPR after three cycles of NACI and radical resection (sintilimab+carboplatin+albumin-bound paclitaxel) (Fig. 8E–H).

## Discussion

The use of ICI therapy is considered as a significant advance in the treatment of operable NSCLC and has been shown to be more effective than conventional chemotherapy. However, its benefits are limited to certain patients due to the lack of comprehensive biomarkers. Consequently, it is crucial to find biomarkers that can predict patients who will derive the greatest benefit from NACI. The majority of the patients in the study had stage III disease and seemed to benefit from the treatment approach with the same level, in terms of MPR percentage, compared to patients with stage I–II disease, highlighting the importance of this therapeutic pattern for patients with more locally advanced disease and worse prognosis.

In addition to assess the efficacy and safety of NACI in real world, our study investigated the pretreatment factors associated with MPR to establish and verify a risk-scoring system. Following a broad analysis of 43 easily accessible clinical-pathological and laboratory parameters, finally, we developed a novel combined model with good discrimination to screen patients who might benefit from therapeutic strategy. In this study, we identified seven factors, including PD-L1, pathology, smoking, PT, NEUT%, P-LCR, and EOS% in the final predictive model. As a classic predictor of immunotherapy, PD-L1 expression levels in the tumor tissue were included in this model. LUSC exhibited superior response rates compared to non-LUSC (MPR 42.1 vs.57.7%). The main histological subgroups of NSCLC, such as LUAD and LUSC, have been found to behave differently in neoadjuvant circumstances, due to different immune microenvironment profiles^37–39^.

In our study, it was very interesting to find another significant correlation between PT and MPR. The coagulation and fibrinolysis processes in patients are often associated with tumor invasion, metastasis, and ultimately poorer outcomes. Hypercoagulability is a sign of a more aggressive phenotype. Higher levels of D-dimer, fibrinogen, and platelet count in the blood are linked to a worse prognosis in NSCLC^40–42^. In summary, PT is a novel independent biomarker for predicting the response to NACI and should be evaluated in future studies to confirm its prognostic significance in operable NSCLC.

Additionally, we found that smoking is an actionable biomarker for predicting MPR with NACI. Similar results have been reported in advanced NSCLC^43–47^. Moreover, in one study, smoking signature performed better than PD-L1 expression in predicting the pathological response in a neoadjuvant cohort, which might be explained by increased TMB and/or MSI resulted from tobacco exposure^48^. Tobacco smoking has recently been demonstrated to induce specific activation of the ADAM12+ Treg subset, which interact with exhausted T cells in the tumor immune environment^49^. Yin *et al*.^50^ study pointed out that after tobacco exposure, patients with a smoking history exhibited higher levels of TLS via the CCL21-dependent mechanism, while serum CCL21 was identified as a reliable biomarker for predicting more benefits from immunotherapy. The findings of current study also validated that smoking history was a promising predictor for MPR to NACI. Smoking status, which is easily available information, could be used as a predictor in clinical practice to select better treatment in the neoadjuvant setting for patients with operable NSCLC.

We found that the results of a few laboratory tests derived from cytometry were also correlated with our endpoint in this cohort, which included NEUT%, P-LCR, and EOS%. The importance of neutrophils in the immune response against cancer has been emphasized in recent years, although much remains unknown about their specific role. A large number of studies have reported that relative or absolute increases in neutrophil counts are associated with negative outcomes in advanced cancers treated with PD-1 inhibitors^51,52^. As we expected, the percentage of neutrophil count maintained its significance in both Logistic and Lasso models. Eosinophils are a minor population of granulocytes that are more frequently explored in asthma and allergic disorders. Nonetheless, eosinophils infiltrate multiple solid tumor types and modulate tumor progression either directly by interacting with tumor cells or indirectly by shaping the tumor microenvironment^53,54^. Across various types of cancer, several authors have demonstrated an association between blood eosinophils and ICI antibodies, however, the data exist in NSCLC was limited. The authors stated that there was a correlation between elevated blood eosinophils and a favorable clinical or radiological outcome^55,56^. To our knowledge, this is the first study to report the role of blood eosinophils in predicting MPR in patients with resectable NSCLC receiving NACI. Clinical data suggests that raised blood eosinophils may reflect a favorable outcome in patients treated with ICIs for advanced NSCLC. Functional studies and more stringent clinical research are needed to further elucidate the role of eosinophils in lung cancer and their potential value as a biomarker. The P-LCR refers to the ratio of platelets with a volume greater than 12 femtolitre (fL) to the total platelet count. The activity and enzymes of larger platelets are more active compared to small platelets, and the release of thrombotic and inflammatory agents from them can lead to specific complications related to diseases^57^. Previous studies have explored the use of platelet parameters to predict complications like diabetic kidney disease in type 2 diabetes, major adverse cardiovascular events in myocardial infarction^58,59^. Although, platelet’s role was involved in immune system and surveillance, platelet indices has not been explored in cancer immunotherapy.

However, attention should be paid to the limitations of the present study. Initially, due to the inherent nature of retrospective study, selection bias was inevitable, although we reported the largest real world cohort to date. Hence, a series of large, multicenter and prospective studies should be conducted to verify our proposed model. Secondly, although 10-fold 200 cross-validations and time-series external validations were used to validate the model, data from other centers were indeed required to check its stability. Thirdly, the endpoint in this study was MPR, which was a surrogate indicator of DFS and OS. We will continue to follow up and look forward to the long-term results.

In conclusion, the nomogram constructed on readily available clinical-pathological characteristics and serum parameters was a simple and cost-effective predictive tool for patients with operable treated with NACI. The combined model achieved an AUC of 0.775 demonstrating a good consistency between the predicted and observed results. Although the efficiency of the proposed model is not sufficient as a direct determinant of MPR, it may help physicians potentially recognize NSCLC who might be sensitive to NACI.

## Ethical approval

This observational cohort study was conducted at a single center and adhered to the ethical guidelines outlined in the Declaration of Helsinki. The study was approved by the Ethics Committee of Beijing Chest Hospital, Capital Medical University (Approval Number: YNLX-2022-015).

## Consent

The study was approved by our institution’s review board (Approval Number: YNLX-2022-015). Due to the observational study design, the ethics committee approved a waiver of written informed consent.

## Sources of funding

This study was supported by the Beijing Municipal Administration of Hospitals Incubating Program and Beijing Municipal Science and Technology Commission, Tongzhou Technology Project (code: PX2024058).

## Author contribution

M.H.: conceptualization, data curation, formal analysis, and writing – original draft; X.L.: formal analysis, methodology, and writing – original draft; H.L.: formal analysis, investigation, and writing – original draft; B.L.: software and supervision; Q.W.: methodology and resources; L.T.: investigation and software; H.L.: supervision and validation; N.C.: resources, visualization, and writing – review and editing; S.H.: supervision and validation; Y.H.: resources and project administration; K.S.: methodology and project administration; C.L.: resources and supervision; H.Z.: conceptualization, methodology, resources, and validation; Z.L.: conceptualization and writing – review and editing.

## Conflicts of interest disclosure

The authors declare no conflicts of interest.

## Research registration unique identifying number (UIN)

1. Name of the registry: Research Registry.
2. Unique identifying number or registration ID: researchregistry9649.
3. Hyperlink to your specific registration (must be publicly accessible and will be checked):https://www.researchregistry.com/browse-theregistry#home/registrationdetails/653dd4a2a2ef180027f90e80/.

## Guarantor

Tongmei Zhang.

## Data availability statement

The corresponding author can provide the datasets used and/or analyzed during the current study upon reasonable request.

## Provenance and peer review

Not applicable.

## Supplementary Material

SUPPLEMENTARY MATERIAL
