# Supplementary material for: Easily applicable predictive score for MPR based on parameters before neoadjuvant chemoimmunotherapy in operable NSCLC: a single-center, ambispective, observational study
Source: Int J Surg. 2024 Jan 23;110(4):2275–87. doi: 10.1097/JS9.0000000000001050 (PMC11020048; doi:10.1097/JS9.0000000000001050)

**Caption for Supplementary Material**

1. Table S1
2. Table S2
3. Table S3
4. Table S4

5. Figure S1

6. Figure S2

7. Figure S3

8. Figure S4

9. Figure S5

**Table S1**

| Variables(median [IQR]) | Groups | |  |
| --- | --- | --- | --- |
|  | Non-irAE | irAE | P |
| Cyfra21-1 | 5.61(3.15-16.84) | 5.45(3.99-8.33) | 0.87 |
| LDH | 168.00(149.50-191.00) | 160.00(139.50-185.25) | 0.12 |
| CRP | 5.87(1.84-19.43) | 5.84(1.60-15.26) | 0.67 |
| CEA | 3.19(2.00-6.61) | 3.19(2.24-5.13) | 0.98 |
| PT | 11.30(10.80-11.90) | 11.40(10.80-11.90) | 0.61 |
| INR | 0.98(0.93-1.03) | 0.99(0.93-1.02) | 0.55 |
| PT% | 84.70(77.40-96.90) | 88.00(80.20-105.75) | 0.12 |
| PTR | 0.98(0.93-1.03) | 0.99(0.94-1.02) | 0.46 |
| APTT | 28.45(25.88-31.42) | 28.00(25.78-30.93) | 0.85 |
| FIB(S) | 6.70(5.60-7.70) | 6.80(5.60-8.78) | 0.30 |
| FIB | 3.46(2.84-4.68) | 3.44(2.80-4.35) | 0.62 |
| TT | 18.80(18.00-19.40) | 18.80(17.90-19.60) | 0.56 |
| D-Dimer | 0.41(0.25-0.63) | 0.45(0.28-0.64) | 0.45 |
| WBC | 7.25(6.44-8.50) | 7.86(6.36-10.03) | 0.63 |
| LY% | 24.10(19.22-27.58) | 21.95(19.63-31.73) | 0.55 |
| M0N0% | 5.95(5.43-6.85) | 6.20(5.60-6.85) | 0.40 |
| NEUT% | 67.05(62.10-73.70) | 64.55(56.53-71.13) | 0.30 |
| RBC | 4.49(4.07-4.75) | 4.50(4.42-4.93) | 0.78 |
| HGB | 137.5(127.00-148.25) | 136.00(130.75-148.50) | 0.66 |
| HCT | 40.25(37.00-42.53) | 40.60(38.48-44.10) | 0.59 |
| MCV | 89.15(86.98-94.10) | 89.45(86.70-91.53) | 0.28 |
| MCH | 30.6(29.60-32.05) | 29.80(29.38-30.95) | 0.40 |
| MCHC | 344.00(333.00-350.50) | 338.5(327.25-342.00) | 0.80 |
| RDW-CV | 12.75(12.23-13.60) | 13.15(12.58-13.23) | 0.79 |
| PLT | 209.00(184.00-278.00) | 234.00(174.25-280.50) | 0.60 |
| MPV | 10.30(9.43-11.65) | 10.05(9.35-12.08) | 0.10 |
| P-LCR | 28.10(20.18-38.58) | 21.10(17.30-38.33) | 0.26 |
| PDW | 12.35(10.70-15.98) | 12.30(9.73-15.18) | 0.82 |
| EOS% | 1.90(1.10-2.75) | 3.05(1.73-6.22) | 0.14 |
| BASO% | 0.50(0.30-0.69) | 0.50(0.20-0.675) | 0.17 |
| RDW-SD | 41.95(39.98-45.25) | 41.85(41.18-44.13) | 0.45 |
| NLR | 2.56(1.94-3.62) | 2.45(1.78-3.36) | 0.38 |
| LMR | 3.93(2.85-5.17) | 3.97(3.26-5.15) | 0.44 |
| PLR | 139.76(103.55-187.18) | 131.34(90.76-162.31) | 0.23 |
| Table S2 The correlation between the irAE and the values of blood markers. AE adverse effect; LDH, lactate dehydrogenase; CRP,C-reactive protein;CEA,carcinoembryonic antigen; PT,prothrombin time; INR, international normalized ratio;PT%, prothrombin percentage;PTR,prothrombin ratio;APTT,partial thromboplastin time; FIB(S), fibrinogen time;FIB,fibrinogen content;TT,thrombin time;WBC,white blood cell; LY%, lymphocyte percentage; M0N0%, monocyte percentage;NEUT%, neutrophilic granulocyte percentage; RBC,red blood cell; HGB, hemoglobin; HCT, hematocrit;MCV,mean corpuscular volume;MCH,mean corpuscular hemoglobin; MCHC,mean corpuscular hemoglobin concentration;RDW-CV,red blood cell distribution-coefficient of variation; PLT,platelet; MPV,mean platelet volume; P-LCR,platelet large cell ratio;PDW,platelet distribution width; EOS%, eosinophilic cells percentage;BASO%,basophilic cells percentage;RDW-SD,red cell distribution width-standard deviation; NLR, neutrophil-to-lymphocyte ratio;LMR,lymphocyte-to-monocyte ratio;PLR, platelet-to- lymphocyte ratio. | | | |

**Table S2**

|  | IIIA(194) | | | | | | IIIB(52) | | | |
| --- | --- | --- | --- | --- | --- | --- | --- | --- | --- | --- |
| Characteristics | T1N2(n=15) | T2N2(n=58) | T3N1`(n=7) | T4N0(n=16) | T4N1n=(8) |  | T3N2(n=27) | T4N2(n=24) | T2N3(n=1) |  |
| Extent of resection |  |  |  |  |  |  |  |  |  |  |
| lobectomy | 12(80.0%) | 43(74.1%) | 5(71.4%) | 13(81.3%) | 7(87.5%) | 0.61 | 17(77.8%) | 21(70.8%) | 1(100.0%) | 0.92 |
| bilobectomy | 3(20.0%) | 11(19.0%) | 1(14.3%) | 2(12.5%) | 0(0%) |  | 4(18.5.0%) | 5(16.7%) | 0(0%) |  |
| pneumonectomy | 0(0%) | 4(6.9%) | 1(14.3%) | 1(6.2%) | 1(12.5%) |  | 3(3.7%) | 1(12.5%) | 0(0%) |  |
| Type of surgery |  |  |  |  |  |  |  |  |  |  |
| thoracotomy | 11(73.3%) | 30(51.7%) | 4(57.1%) | 9(56.2%) | 5(62.5%) | 0.67 | 15(55.6%) | 12(50.0%) | 0(0%) | 0.53 |
| VATS | 4(26.7%) | 28(48.3%) | 3(42.9%) | 7(43.8%) | 3(37.5%) |  | 12(44.4%) | 12(50.0%) | 1(100.0%) |  |
| Pathological evaluation |  |  |  |  |  |  |  |  |  |  |
| MPR | 8(53.3%) | 31(53.4%) | 3(42.9%) | 10(62.5%) | 5(62.5%) | 0.91 | 13(48.1%) | 12(50.0%) | 0(0%) | 0.92 |
| mon-MPR | 7(46.7%) | 27(46.6%) | 4(57.1%) | 6(37.5%) | 3(37.5%) |  | 14(51.9%) | 12(50.0%) | 1(100.0%) |  |
| Table S3: Treatment outcomes in TNM III stage. VATS,video-assisted thoracoscopic surgery; MPR, major pathological response. | | | | | | | | | | |

**Table S3**

| Characteristic | OR (95%CI) | P |
| --- | --- | --- |
| Age | 1(0.96-1.04) | 0.89 |
| Gender (male vs female) | 2.77(1.19-6.46) | 0.02 |
| Smoking  (never vs current/former) | 0.45(0.23-0.88) | 0.02 |
| Pathological type  (LUSC vs non-LUSC) | 1.88(1.01-3.48) | 0.05 |
| cTNM stage (IIIA vs I-II) | 0.95(0.48-1.88) | 0.89 |
| cTNM stage (IIIB vs I-II) | 0.73(0.33-1.59) | 0.42 |
| Neoadjuvant cycles (>2 vs≤2) | 0.99(0.57-1.72) | 0.97 |
| BMI | 0.95(0.87-1.03) | 0.22 |
| PD-L1 expression | 1.01(1.01-1.02) | <0.001 |
| Cyfra21-1 | 1.01(0.99-1.02) | 0.55 |
| LDH | 1(0.99-1) | 0.74 |
| CRP | 1(0.99-1.02) | 0.53 |
| CEA | 1(1-1) | 0.88 |
| PT | 1.62(1.19-2.21) | <0.001 |
| INR | 1(0.98-1.02) | 0.87 |
| PT% | 0.99(0.98-1) | 0.02 |
| PTR | 147.13(4.4-4917.93) | 0.01 |
| APTT | 1.07(1.01-1.15) | 0.03 |
| FIB(S) | 1.05(0.98-1.13) | 0.13 |
| FIB | 1.12(0.89-1.43) | 0.34 |
| TT | 0.99(0.83-1.18) | 0.89 |
| D-Dimer | 1.23(0.78-1.95) | 0.36 |
| WBC | 0.99(0.87-1.12) | 0.86 |
| LY% | 1.04(1-1.07) | 0.04 |
| M0N0% | 1.07(0.91-1.25) | 0.43 |
| NEUT% | 0.96(0.93-0.99) | 0.01 |
| RBC | 1.14(0.67-1.93) | 0.63 |
| HGB | 0.99(0.98-1.01) | 0.54 |
| HCT | 0.99(0.94-1.05) | 0.8 |
| MCV | 0.96(0.9-1.01) | 0.13 |
| MCH | 1.01(0.99-1.04) | 0.23 |
| MCHC | 0.99(0.97-1) | 0.09 |
| RDW-CV | 1.24(0.96-1.58) | 0.09 |
| PLT | 1(1-1) | 0.46 |
| MPV | 1.22(0.95-1.58) | 0.12 |
| P-LCR | 1.05(1.01-1.08) | 0.01 |
| PDW | 1.11(0.99-1.24) | 0.06 |
| EOS% | 1.25(1.06-1.48) | 0.01 |
| BASO% | 2(0.72-5.58) | 0.18 |
| RDW-SD | 0.99(0.93-1.05) | 0.72 |
| NLR | 0.86(0.73-1.01) | 0.06 |
| PLR | 0.98(0.94-1.02) | 0.33 |
| LMR | 1.13(0.93-1.36) | 0.21 |
| Table S4 Univariate logistic regression for major pathological response of all included patients.LUSC,lung squamous cell carcinoma; PD-1, programmed death-1; BMI,body mass index; LDH,lactatedehydrogenase; CRP,C-reactive protein;CEA,carcinoembryonic antigen; PT,prothrombin time; INR, international normalized ratio;PT%,prothrombin percentage;PTR,prothrombin ratio;APTT,partial thromboplastin time;FIB(S), fibrinogen time;FIB,fibrinogen content;TT,thrombin time;WBC,white blood cell; LY%, lymphocyte percentage; M0N0%, monocyte percentage;NEUT%,neutrophilic granulocyte percentage; RBC,red blood cell; HGB, hemoglobin;HCT, hematocrit;MCV,mean corpuscular volume;MCH,mean corpuscular hemoglobin; MCHC,mean corpuscular hemoglobin concentration;RDW-CV,red blood cell distribution-coefficient of variation; PLT,platelet; MPV,mean platelet volume ;P-LCR,platelet large cell ratio;PDW,platelet distribution width; EOS%, eosinophilic cells percentage;BASO%,basophilic cells percentage;RDW-SD,red cell distribution width-standard deviation; NLR, neutrophil-to-lymphocyte ratio;LMR,lymphocyte-to-monocyte ratio;PLR,platelet-to-lymphocyte ratio. | | |

**Table S4**

| Indicators | Model dataset | Mean of cross-validation dataset | External validation dataset |
| --- | --- | --- | --- |
| AUC | 0.775 | 0.75 | 0.835 |
| R2 | 0.304 | 0.01 | 0.419 |
| D | 0.253 | 0.08 | 0.332 |
| U | −0.010 | 0.02 | -0.050 |
| Brier | 0.191 | 0.21 | 0.154 |
| Emax | 0.046 | 0.31 | 0.099 |
| Eavg | 0.016 | 0.14 | 0.051 |
| Table S5: Cross validation was used to evaluate the repeatability of the model.AUC, area under the curve. | | | |

**Figure S1:** The flowchart of screening criterion in this study.


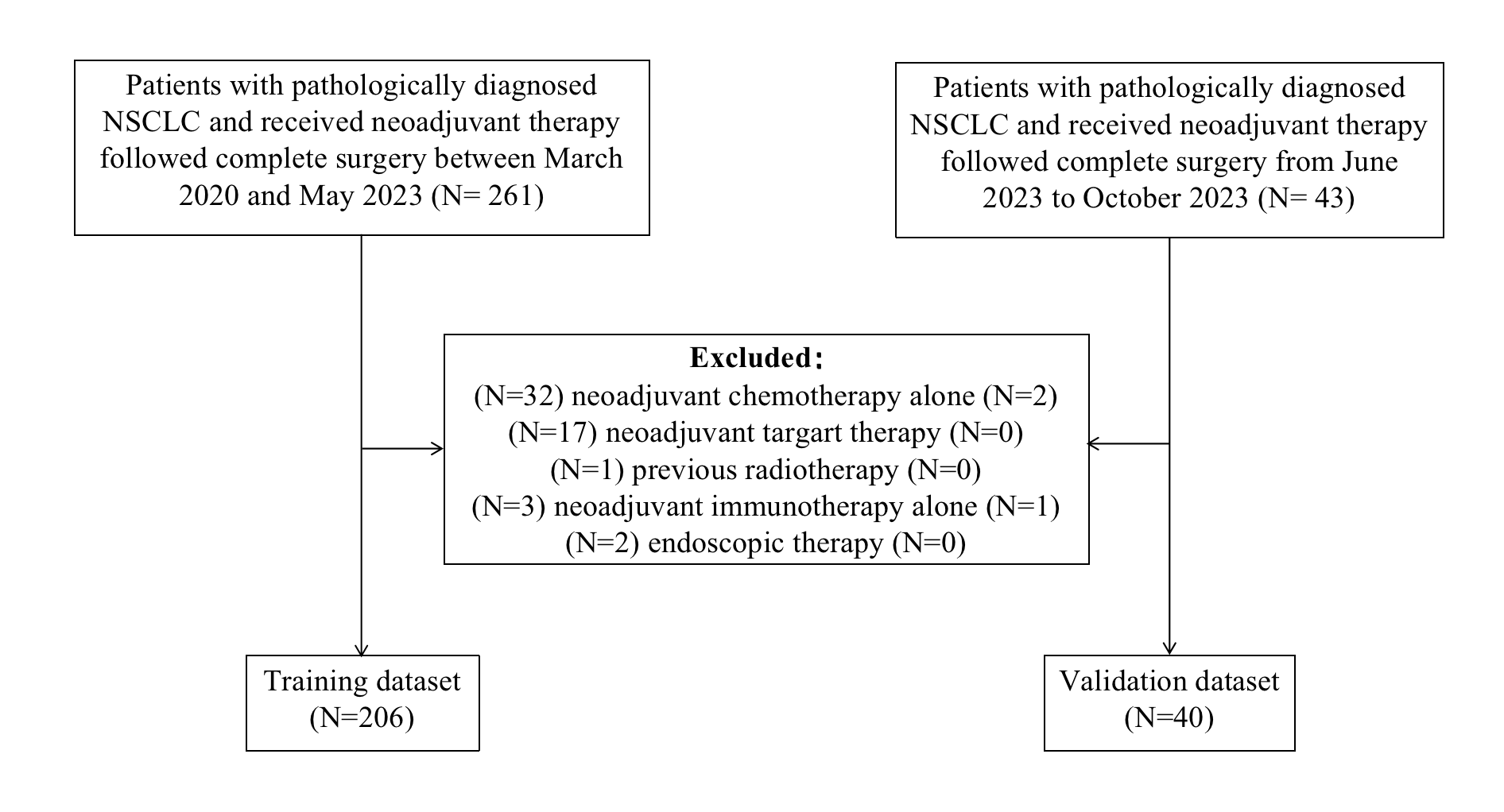


**
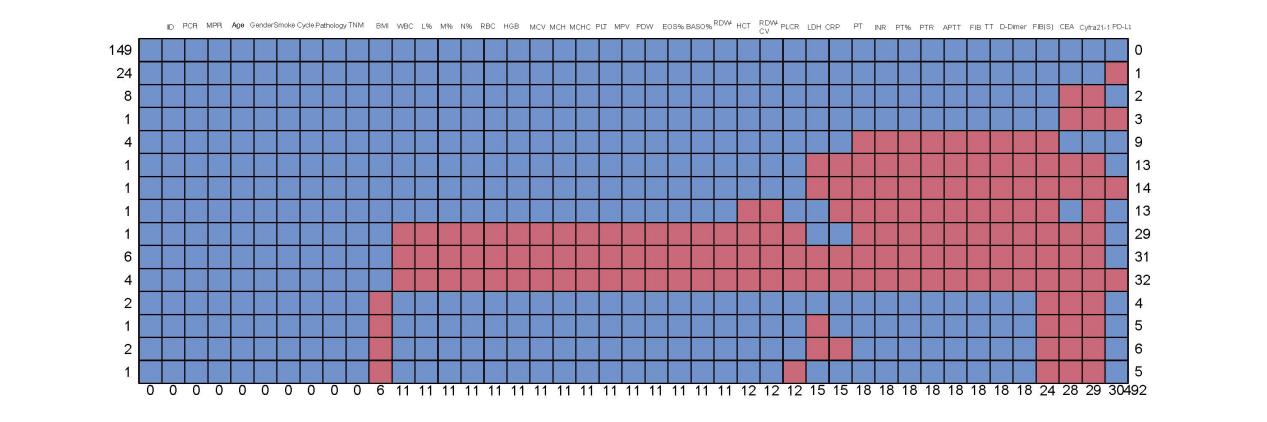
Figure S2:** Demonstration of baseline data integrity.

**
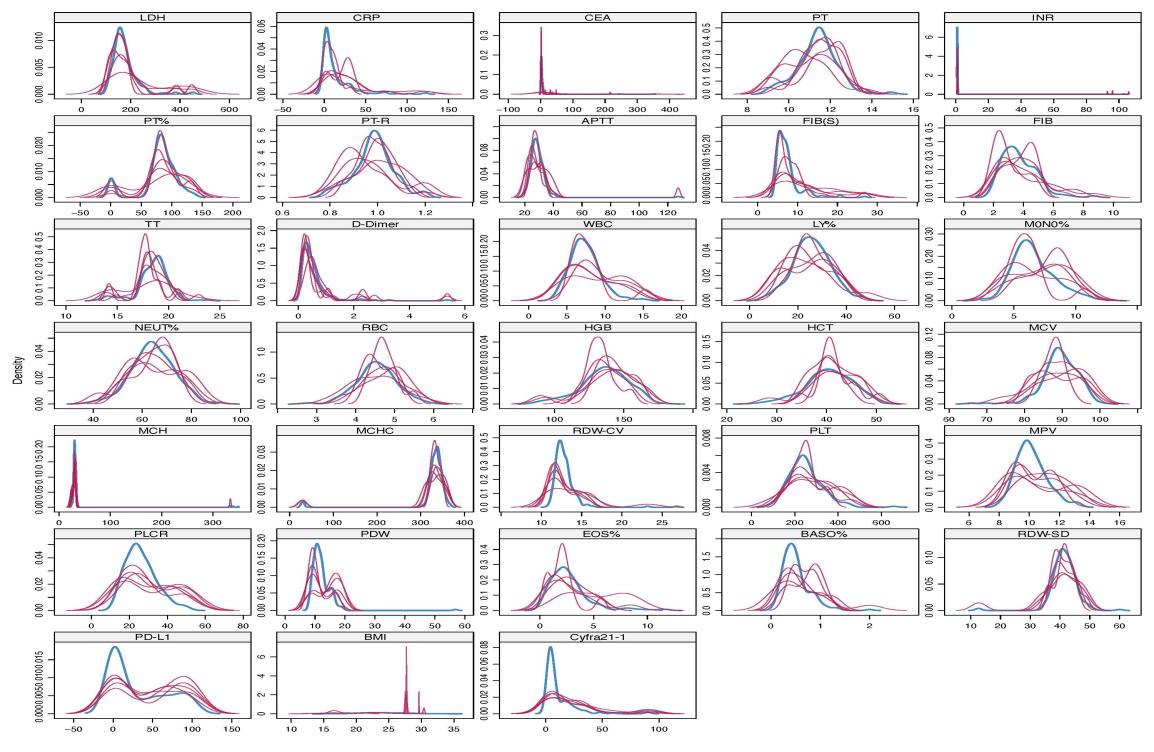
Figure S3:** The density plots of data after multiply imputed using a predictive mean matching method.

**Figure S4:** Predictive model discriminatory power in objective response rate, a. ROC curve; b. Histogram of TPS comparison between ORR and non-ORR groups.


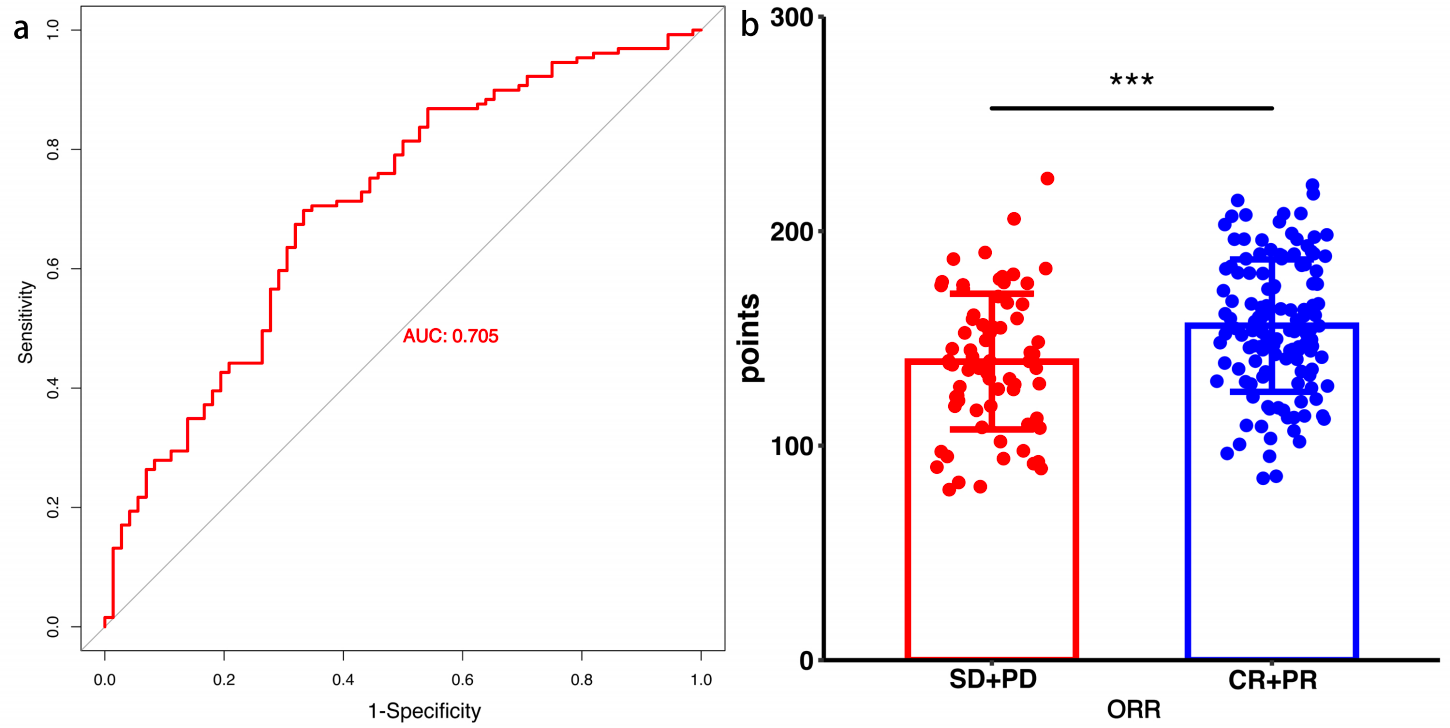


**Figure S5:** The MPR percentage across increasing quartiles of TPS


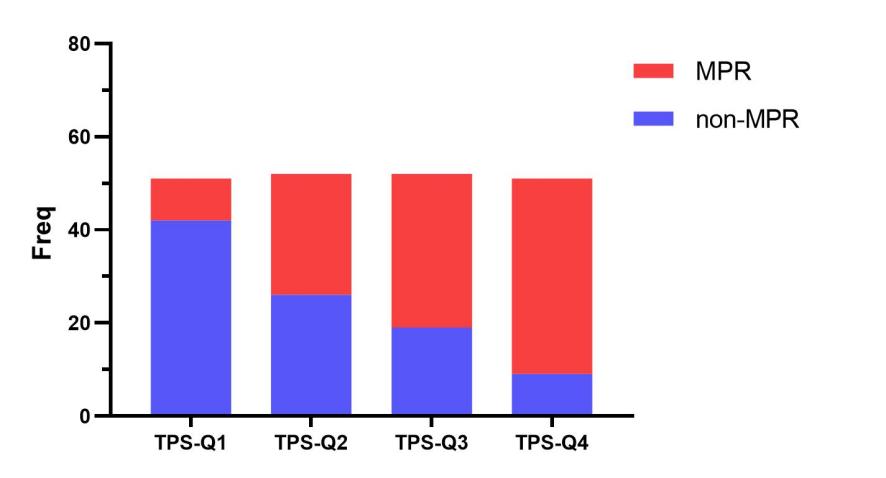

Supplement: SUPPLEMENTARY MATERIAL [file js9-110-2275-s002.docx]
